# Evidence for a palaeo-subglacial lake on the Antarctic continental shelf

**DOI:** 10.1038/ncomms15591

**Published:** 2017-06-01

**Authors:** Gerhard Kuhn, Claus-Dieter Hillenbrand, Sabine Kasten, James A. Smith, Frank O. Nitsche, Thomas Frederichs, Steffen Wiers, Werner Ehrmann, Johann P. Klages, José M. Mogollón

**Affiliations:** 1 Alfred-Wegener-Institut Helmholtz-Zentrum für Polar- und Meeresforschung, Department of Geosciences, 27568 Bremerhaven, Germany; 2 British Antarctic Survey, High Cross, Madingley Road, Cambridge CB3 0ET, UK; 3 Lamont-Doherty Earth Observatory of Columbia University, Palisades, New York 10964, USA; 4 Department of Geosciences, University of Bremen, 28359 Bremen, Germany; 5 Institute for Geophysics and Geology, University of Leipzig, 04103 Leipzig, Germany; 6 Department of Earth Sciences–Geochemistry, Faculty of Geosciences, Utrecht University, 3584 CC Utrecht, The Netherlands

## Abstract

Subglacial lakes are widespread beneath the Antarctic Ice Sheet but their control on ice-sheet dynamics and their ability to harbour life remain poorly characterized. Here we present evidence for a palaeo-subglacial lake on the Antarctic continental shelf. A distinct sediment facies recovered from a bedrock basin in Pine Island Bay indicates deposition within a low-energy lake environment. Diffusive-advection modelling demonstrates that low chloride concentrations in the pore water of the corresponding sediments can only be explained by initial deposition of this facies in a freshwater setting. These observations indicate that an active subglacial meltwater network, similar to that observed beneath the extant ice sheet, was also active during the last glacial period. It also provides a new framework for refining the exploration of these unique environments.

## Abstract (web summary)

While a number of subglacial lakes have been discovered beneath the modern Antarctic ice sheet, little is known regarding their prevalence during past glaciations. Here, Kuhn *et al*. present sedimentological evidence for an active palaeo-subglacial lake in Pine Island Bay during the last glaciation.

Over the past decade, satellite observations and radar measurements revealed that an active, partially interconnected subglacial hydrological system of >379 lakes exists beneath the Antarctic Ice Sheet^1^. The periodic filling and draining of some of these subglacial lakes^2^,^3^ has been associated with transient acceleration of ice streams^4^, demonstrating that they are a vital component of ice-sheet dynamics, which control ice sheet mass balance and hence sea-level fluctuations^5^. However, with limited access to these unique environments, significant questions remain about the influence of subglacial water storage and transfer and its influence on ice-sheet stability and the ability of subglacial lakes to support life^6^.

With the exception of direct access to the subglacial Lake Vostok (accreted lake ice only)^7^ and the ice-sheet grounding zone at subglacial Lake Whillans^8^,^9^,^10^, the subglacial hydrological system in Antarctica^11^ has been explored using remote sensing and numerical models with the latter predicting the number of potential lakes to >12,000 (ref. 12). However, recent model development has largely outpaced the acquisition of geological data required for their validation^13^. Consequently, climate models of ice-sheet responses to global change, and hence predictions of future sea-level rise, remain incomplete without data and a reliable algorithm for simulating subglacial lake formation and drainage and its influence on ice dynamics^14^,^15^.

One alternative to the logistical and technical challenges of *in situ* lake drilling^16^ that can address some of the objectives of subglacial lake exploration is the investigation of subglacial lakes that once existed beneath former ice sheets^17^. This offers significant advantages because we can obtain a more complete understanding of the bed properties (that is, geology and topography) and can sample and analyse the sediments without complex logistics. Modelling suggests that subglacial lakes were more widespread in Antarctica during the last glacial maximum (LGM; 23–19 ka before present (BP)) when grounded ice had extended farther onto the continental shelf^12^. Furthermore, geomorphological findings from the deep, predominantly bedrock-floored inner shelf indicate the past existence of large-scale subglacial drainage networks, possibly including lakes that stored water and evacuated it to the ice-sheet margin^18^. On the basis of modelling work it has been argued that outburst floods from these palaeo-lakes were associated with surging of ice streams and the erosion of the channels now observed^19^. However, despite recent drilling through to Lake Whillans, an ephemeral lake under the lightly grounded down-stream part of Whillans Ice Stream^8^, the criteria for the identification of former subglacial lakes remain largely theoretical^6^,^17^. Coring at Lake Whillans recovered a soft diamicton, interpreted as a subglacial till deposited during intermittent ice-stream grounding phases that followed lake drainage events, but no subglacial lake sediments^9^.

## Results

### Lake sediment facies and lithostratigraphic units

**Figure 1:**
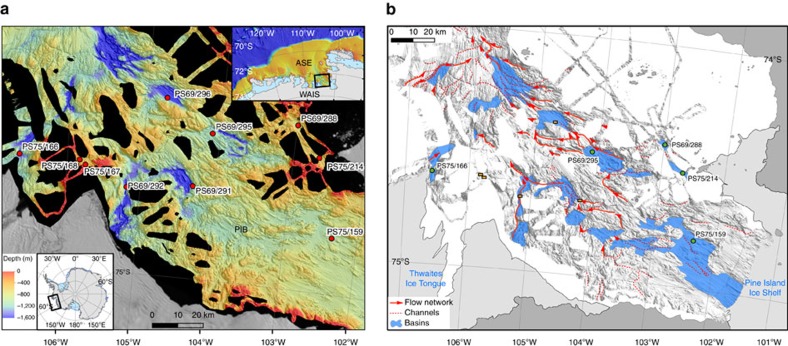
Location map. (**a**) Bathymetric map of Pine Island Bay (PIB) on the inner continental shelf of the Amundsen Sea Embayment (ASE; see inset on top) with the locations of the investigated sediment cores (black areas=data gaps; grey areas=glacial ice). Inset map at bottom shows the ASE within the wider context of Antarctica (WAIS, West Antarctic Ice Sheet). (**b**) Map of potential palaeo-subglacial lakes (blue) inferred from the similarity of geomorphological features in PIB with dimensions of subglacial lakes under the modern Antarctic Ice Sheet^1^. Green dots mark core sites where sediments with lithofacies and/or pore water chloride concentrations, which are indicative of a palaeo-subglacial lake setting were recovered. Brown rectangles mark locations of cores without very low Cl^−^ concentrations in grounding zone proximal sediments at core base (map modified from Nitsche *et al*.^18^; dark grey areas=grounded ice).

**Figure 2:**
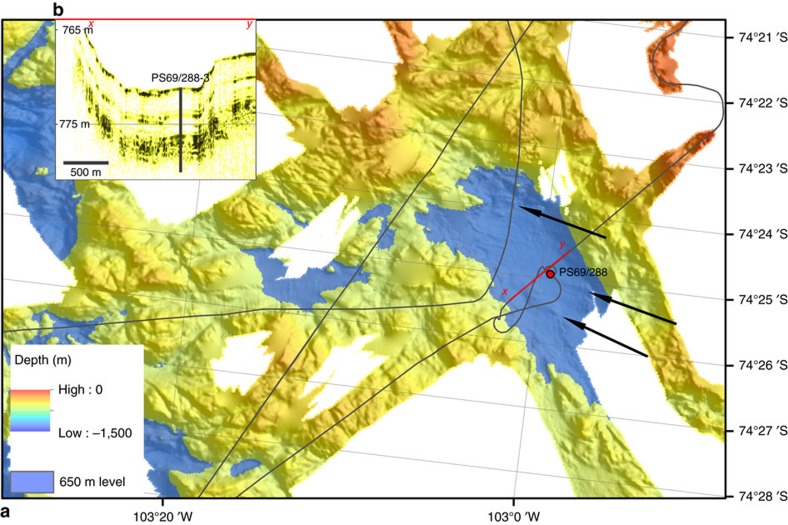
Potential subglacial lake basin at site PS69/288. (**a**) The basin floor at site PS69/288 has a modern water depth of 750–800 m and the surrounding sill has a water depth of 500–650 m (650 m marked blue). Drumlinoid features (indicated by black arrows) of unknown age are observed in the centre of the basin documenting that grounded ice was present there^18^, probably during the LGM like in the wider region^20^,^21^,^22^ (Fig. 1a,b). Dark grey lines are ANT-XXIII/4 ship tracks. (**b**) Red line indicates a PARASOUND^18^ sub-bottom profile displayed in inset figure (b).

**Figure 3:**
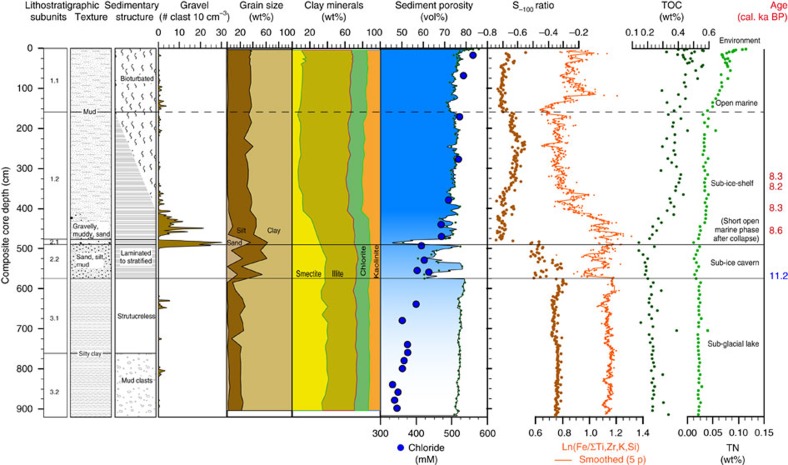
Lithostratigraphic units and palaeo-environmental proxies in core PS69/288. Sedimentological, geophysical and geochemical parameters are from left to right: Lithostratigraphic units, texture, sedimentary structures, gravel counts, grain size distribution, clay mineralogy, sediment porosity from core-logger measurements (line) and discrete samples (small dark green dots), chloride concentration in pore water (down-core shading of porosity curve from dark to light blue highlights decreasing salinity derived from decreasing chloride concentration), rock magnetic S_−100_ ratio, Fe ratio, TOC contents (total organic carbon), TN contents (total nitrogen). Lithostratigraphic Subunits 3.2 and 3.1 were deposited in a subglacial lake setting, Subunits 2.2 and 2.1 reflect the transition from a subglacial lake to a sub-ice shelf cavity, while Subunits 1.2 and 1.1 demonstrate increasing (open) marine influence on sedimentation. Additional support for a subglacial lake setting of Subunits 3.2 and 3.1 comes from increased concentrations of the minerals haematite and goethite and/or fine-grained (titano-)magnetites/maghemites, which are evident from both rock magnetic parameters (S_−100_ and S_−300_ (not shown here) ratios; see Methods) and elevated iron concentrations (Ln(Fe/∑Ti,Zr,K,Si)) from XRF core scanner measurements (Methods). The high concentrations of these iron-oxide minerals suggest Fe dissolution and Fe deposition in an aquatic environment with multiple oxidation–reduction reactions, typical for subglacial hydrological systems with high melt-water content^54^. Ages see Table 2 (blue 11.2 ka from core PS75/214).

**Figure 4:**
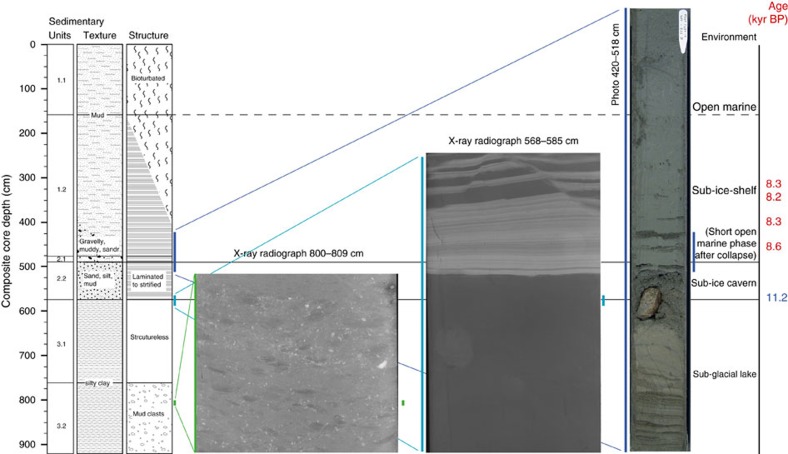
X-radiographs and photograph from core PS69/288-3. Features characteristic for the subglacial lake lithofacies of Subunits 3.2 (mud clasts) and 3.1 (structureless silty clay) and for the brackish lithofacies of Subunit 2.2 (fine scale laminations) are best seen in X-radiographs. Sand and silt-layer deposition in Subunit 2.2 can be attributed to enhanced ocean-driven melt out of subglacially transported debris at the grounding line surrounding the subglacial cavern and/or to increased current velocities by tidal flows.

Here we present a robust data set for subglacial lake sedimentation on the Antarctic shelf during the last glacial period, based on geochemical, geophysical and sedimentological analyses on a marine sediment core from site PS69/288 (74° 24.94′ S, 102° 59.48′ W, 772 m water depth; Table 1). The 9.18 m-long sedimentary succession (composed of gravity corer (GC) and giant box corer (GBC), core length of GC is 9.12 in Table 1, see Methods) was retrieved from a small bedrock basin in inner Pine Island Bay, Amundsen Sea, in an area where previous geomorphological work suggested the former existence of a network of subglacial lakes and interconnecting channels^18^ (Figs 1 and 2). The recovered sedimentary succession consists of three lithostratigraphic units recording the transition from a subglacial lake (Unit 3) via a brackish sub-ice cavity setting (Unit 2) to a distal sub-ice shelf and seasonal open-marine environment (Unit 1) (Figs 3 and 4). We analysed chloride ion concentration, a reliable proxy for salinity (Methods), in pore water samples of core PS69/288-3. The low chloride concentrations measured in the fine-grained sediments of basal Unit 3 are interpreted to indicate freshwater conditions during its deposition. Alternative processes to explain the low chloride concentrations, i.e. clay mineral dewatering or opal dehydration, can be ruled out because they require temperatures in excess of 80 and 55 °C (ref. 20), respectively, which are not possible in such a thin sediment drape.

**Figure 5:**
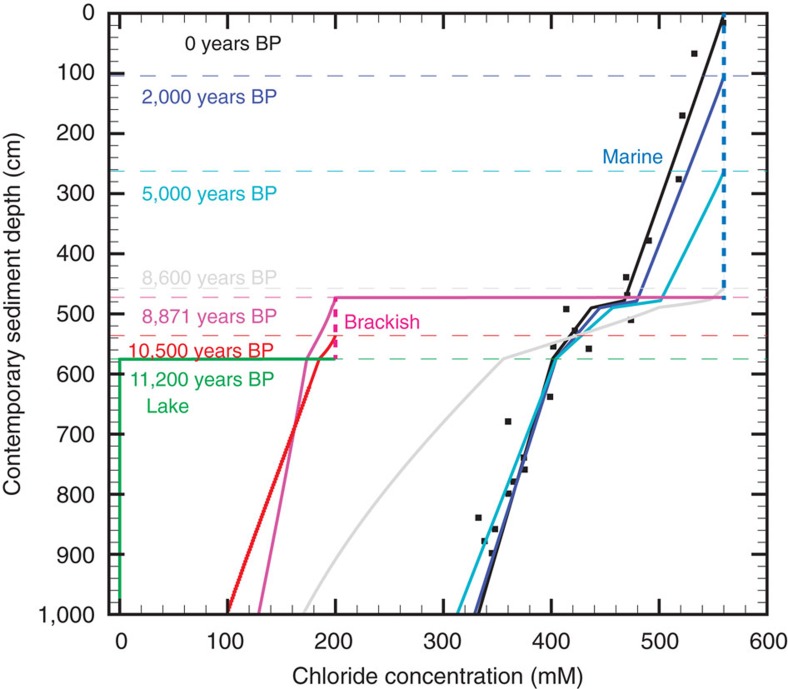
Diffusion-advection model for site PS69/288 describing the evolution of chloride concentrations in pore water through time. Modelled profiles of chloride concentrations in pore water are given for 11,200 years BP (green), 10,500 years BP (red), 8,871 years BP (pink), 8,600 years BP (grey), 5,000 years BP (light blue), 2,000 years BP (dark blue) and 0 years BP (black). The modelled modern profile (=0 years BP) matches the measured chloride concentrations in the core (black squares). Chloride concentrations in the bottom water at site PS69/288 must have been close to 0 mM (=fresh water chloride concentration) during the deposition of the subglacial lake sediments of Subunits 3.2 and 3.1 (green profile), previous to the limnic–brackish transition during deposition of Unit 2 at 11,200 cal. yr BP. The deposition of Unit 2 from 11,200 to 8,900 cal. yr BP (red and pink profiles) was characterized by brackish conditions with chloride concentrations of 200 mM (red dashed line). This phase was followed by a marine stage with chloride concentrations of 560 mM (blue dashed line) that coincided with the deposition of Subunits 1.2 and 1.1 and lasted from 8,900 cal. yr BP until the present day (grey, light blue, dark blue and black profiles) (see also animated graphic in Supplementary Movie 1). For details, see Methods.

Exchange between fresh pore water and seawater must have started as soon as the core site became connected to the ocean. To test different scenarios for the seawater incursion (that is, to investigate whether and when a phase with brackish conditions occurred), we applied diffusive-advective modelling of the chloride concentration gradient over time, thereby using previously published AMS ^14^C ages for core PS69/288-3 (ref. 21) (Table 2) and the regional glacial history^21^,^22^ as chronological constraints (Fig. 5). A distinct lithofacies characterizing the subglacial lake sediments of the purely terrigenous Unit 3 comprises a basal Subunit 3.2 consisting of gravel-sized mud clasts and isolated gravel grains embedded in a silty clay matrix, and an overlying Subunit 3.1 consisting of structureless silty clay (Figs 3 and 4). Unit 3 results from the melt-out of predominantly fine-grained detritus, frozen mud clasts and gravel grains from the base of the ice that formed the roof of the lake cavity, and its subsequent settling through a calm, suspension rich water column. We infer a last glacial period age for the subglacial lake based on the regional glacial history^21^,^22^. The largely homogenous and fine-grained composition of Unit 3 indicates calm hydrologic conditions with limited flow into or out of the lake (Figs 3 and 4). The observed low chloride pore water concentrations suggest deposition in freshwater to brackish environments and are occasionally associated with similar grounding zone proximal lithofacies in several other cores collected from basins in Pine Island Bay (Fig. 1, Table 1).

**Figure 6:**
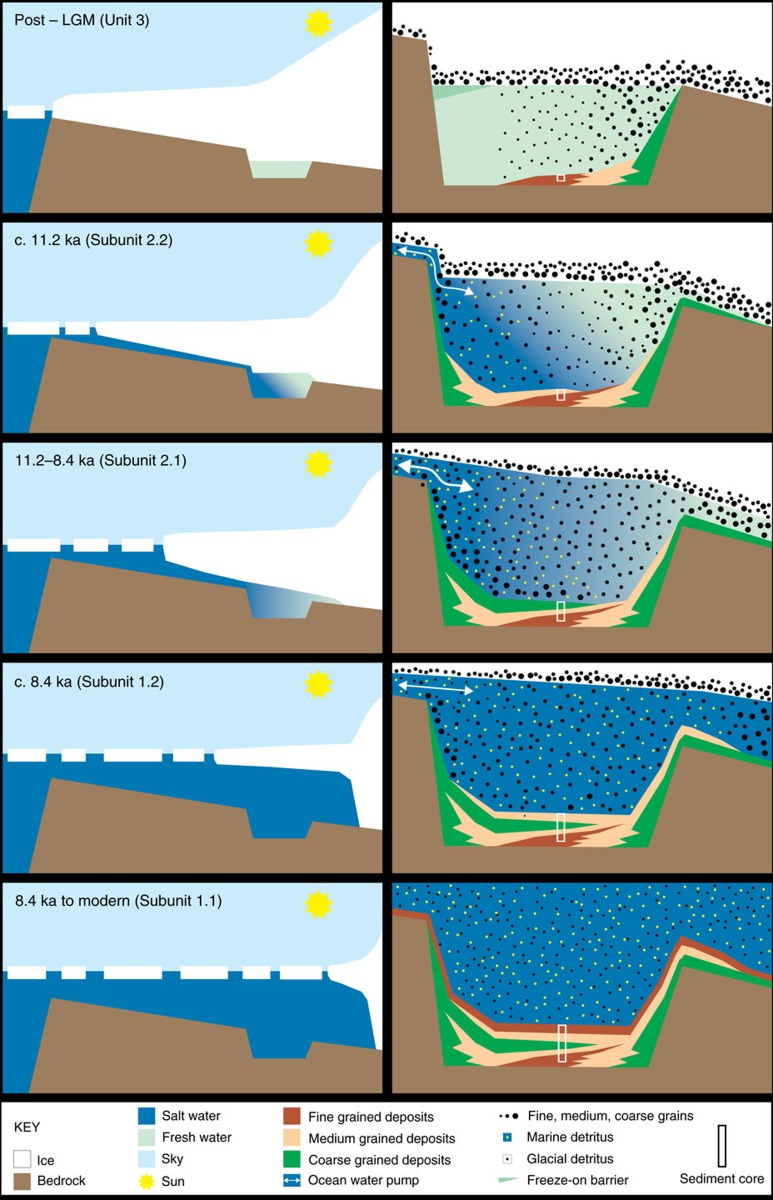
Schematic illustration of the post LGM to modern history of the subglacial lake in the Amundsen Sea Embayment. Profiles over the Amundsen Sea Embayment (ASE) (left panels) and the lake basin (right panels) (South is to the right). Evolution of sedimentary units and subunits from top to bottom.

The succession above Unit 3 at site PS69/288 documents an initial increase and subsequent decrease in transport energy. Unit 2 directly overlies Unit 3 and also comprises purely terrigenous sediments (Fig. 3). Subunit 2.2 consists of horizontal, planar, internally laminated sandy and silty layers alternating with homogenous mud (Fig. 4). The content of the clay mineral smectite throughout Subunit 2.2 is nearly identical to that of Unit 3, thus indicating a very similar sediment provenance and suggesting that the lake was still largely enclosed by grounded ice at that time. However, given the chronological constraints for site PS69/288 (Table 2), our diffusive modelling approach can reproduce the modern gradient of down-core pore water chloride concentrations only, if Subunit 2.2 was deposited in a lake with a minor seawater component (Fig. 5). This finding reveals that at least an episodic, possibly tidal, connection of the subglacial cavity to the ocean had been established at this time, creating an environment comparable to the modern subglacial Lake Whillans in the Ross Sea^8^,^9^,^10^. The post-LGM deglaciation history of Pine Island Bay suggests a minimum age of 11.2 cal. ka BP for the first establishment of a lake–ocean connection^21^,^22^ (Fig. 6). Deposition of the sediments of Subunit 2.2 in an environment with higher energy is evidenced by its fine sand and silt layers that can be attributed to enhanced ocean-driven melt-out of subglacially transported debris at the lake rim as well as ice decoupling from the bed and increase in tidal water exchange to the sub-ice cavity.

The widening of the lake cavity to a sub-ice shelf cavity is indicated by the crudely stratified gravelly muddy sand of Subunit 2.1. The smectite content in this subunit is lower than in the underlying sediments, suggesting increased mixing with fine-grained terrigenous detritus supplied by ocean currents from a wide area in Pine Island Bay^23^. The increasing marine influence within Subunit 2.1 is also reflected by higher chloride concentrations in the pore water (Figs 3 and 5). The coarse texture of the subunit indicates deposition from high-energy flows, probably grain flows, triggered by tidal pumping and intensified oceanic melting at the palaeo-ice stream grounding line which must have retreated landward of site PS69/288 at this time (Fig. 6). Furthermore, the ice sheet must have been close to buoyancy during this phase to allow inflow or exchange with the open ocean. This situation is analogous to the modern Totten Glacier in East Antarctica where seawater penetrates deep into the grounding zone through a narrow (ca. 5 km wide) localized trough^24^.

The subsequent decrease in transport energy at site PS69/288 is documented by deposition of Unit 1 (Fig. 3), whose lower, purely terrigenous Subunit 1.2 (no diatom frustules were found) consists of horizontal, planar silty to sandy layers embedded in a muddy matrix (Fig. 4). Both the coarse-grained layers and scattered, gravelly pebbly grains that represent melt-out of glaciogenic debris from the base of an ice shelf, decrease in number towards the top of this subunit, and we infer that this indicates the continued landward retreat of the palaeo-ice stream grounding line^25^. The lower parts of Subunit 1.2 together with Unit 2 sediments are characteristic for grounding zone proximal sediments. Furthermore, the concomitant intensification of bioturbation towards the top of Subunit 1.2, which is accompanied by elevated contents of total organic carbon (TOC) and total nitrogen (TN), records increased benthos activity in response to enhanced advection of plankton particles from the open ocean under a retreating ice shelf and/or a growth in phytoplankton production facilitated by a decrease of sea-ice coverage leading to longer seasons of open water. AMS ^14^C dates obtained from calcareous foraminifera in the lower part of Subunit 1.2 provided ages between 8.6 and 8.2 cal. ka BP, with the upper three youngest dates overlapping within error (Table 2). During this period of time, the disintegration of an ice shelf in Pine Island Bay may have triggered major rapid ice-sheet thinning further inland^26^. The transition from a glaciomarine setting dominated by long-term ice coverage to the seasonal open-marine conditions characterizing Pine Island Bay today is documented by the deposition of Subunit 1.1. Its muddy sediments show further increase of marine productivity, which is evident from higher TOC and TN contents, intense bioturbation and the presence of marine diatoms near the core top (Fig. 3).

## Discussion

Subglacial lakes are one of the least accessible and explored environments on Earth’s surface, yet have the capacity to modulate ice-sheet mass balance and support viable microbial ecosystems^10^. In Antarctica they have the potential to influence Southern Ocean geochemical and biological systems^27^. Previously it was speculated that information on the origin and longevity of subglacial lakes is contained within their sedimentary successions^6^ but until now this has remained largely theoretical^17^. Our findings demonstrate the first sedimentological and geochemical evidence of the presence of a subglacial lake on the Antarctic shelf and its transition to a glaciomarine environment during the last deglaciation (Fig. 6). Such lakes provide not only constraints on basal thermal regimes and basal properties of past ice sheets that need to be incorporated in numerical models but also offer an opportunity to refine approaches for subglacial lake exploration from ships. We have demonstrated that pore water salinity, and specifically chloride-ion concentration, together with a distinct subglacial lake sediment facies (structureless silty clay with or without mud clasts) provides a powerful set of tools for establishing the presence of palaeo-subglacial lakes. Chronologically constrained diffusive-advective modelling confirms that low chloride concentrations can only be explained by mixing with a freshwater source, while an associated distinct sediment facies (Unit 3) indicates deposition in an enclosed, lacustrine, low-energy environment rather than any other freshwater influenced setting^28^ (for example, low chloride groundwater flow from below). Consequently, our results refine the criteria for interpreting subglacial lake facies^6^,^17^ that will allow an easier identification of subglacial lakes in the palaeo-record. The consistency between our sediment core data and the distribution of subglacial lakes inferred from previous geomorphological studies^18^ (Fig. 1b) offers an opportunity to advance our understanding of subglacial lakes and their sedimentary archives, which are believed to have been numerous on the Antarctic shelf during the last glacial period^12^.

Ultimately, subglacial lakes provide evidence for a wet-based glacial regime. Our study, which effectively ground-truths previous modelling work, has implications for ice-stream formation and flow, bed lubrication, meltwater drainage and theories about meltwater driven ice-sheet collapse^19^,^29^. Whilst future work is needed to confirm that subglacial lakes form part of an interconnected network (Fig. 1b), which drains meltwater from the ice-sheet interior to its margin, our study provides a robust data set to explore how the geometry and capacity of the subglacial hydrologic system influence ice dynamics when the ice-sheet's substrate and profile are known.

## Methods

### Bathymetry

Bathymetric data (Fig. 1) were compiled from existing multi-beam swath bathymetry data acquired by RV *Polarstern*, RV/IB *Nathaniel B. Palmer,* RRS *James Clark Ross* and IB *Oden* as described in Nitsche *et al*.^18^ and are represented by a grid with 30 m resolution. Figure 1b is a modified version of Fig. 7 in Nitsche *et al*.^18^, which shows potential subglacial lakes inferred from the spatial and geomorphological similarity of bathymetric shelf basins and channels carved into bedrock in inner Pine Island Bay with subglacial lakes under the modern Antarctic Ice Sheet. The specific data covering the basin of this study (Figs 1 and 2) were collected during RV *Polarstern* expeditions ANT-XXIII/4 (ref. 30) and ANT-XXVI/3 (ref. 31) using an Atlas Hydrosweep DS2 system. All data were edited and corrected for sound velocity on board.

Analysis of potential lake sill depths were carried out by systematically adding semi-transparent surfaces representing different depth levels from 500 to 700 m (first in 25 m interval steps, then refined to 5 m steps) using ArcGIS and Global Mapper GIS software. A selection of these steps shows the transition from an enclosed subglacial lake to an open subglacial cavern (Fig. 2, Supplementary Fig. 1 and Supplementary Movie 2).

### Material

Marine sediment cores were recovered from inner shelf basins with GC and GBC on RV *Polarstern* expeditions ANT-XXIII/4 (ref. 30) and ANT-XXVI/3 (ref. 31). Sedimentary facies and low pore water chloride concentrations in five of the cores are indicative of palaeo sub-ice lakes (Table 1, Fig. 1), while the basal units of five other cores are characterised by grounding zone proximal sediments with pore water chloride concentrations typical for seawater. The sedimentary succession at site PS69/288 is a composite record (918 cm below sea floor, cmbsf, total recovery) with the upper part of PS69/288-2 GBC being spliced at 6 cm core depth with the core top of PS69/288-3 GC (upper 6 cm lost during core recovery) collected from the same site. The cores were opened, sampled and analysed at the Alfred Wegener Institute, the British Antarctic Survey, University of Bremen and Leipzig University following standard procedures (see below)^32^. Core photos, X-radiographs, sampling intervals and data measured for this study can be found under Pangaea (doi:10.1594/PANGAEA.873755), with the core depth of the PS69/288 record being given as composite depth.

### Grain size analysis

The amount of gravel (number of grains >2 mm in 10 cm^3^ sediment volume) was determined by counting gravel grains at every 1 cm core depth in the X-radiographs, applying the same method which is used to quantify abundance of ice rafted debris^33^ (Figs 3 and 4). For discrete samples (sampling intervals 10 cm), the gravel (>2 mm) and sand (63 μm - 2 mm) fraction was separated from the silt (2–63 μm) and clay (<2 μm) fractions by wet sieving. Afterwards the silt and clay fractions were separated by settling using the Atterberg method, while the gravel and sand fractions were separated by dry sieving.

### Clay mineralogy

Analyses of the clay mineral composition in the sediments followed standard methods^34^,^35^. We mounted the <2 μm sediment fraction as texturally oriented aggregates and X-rayed the slides with a Rigaku MiniFlex system (CoKα radiation; 30 kV; 15 mA) in the range 3–40° 2Θ, with a step size of 0.02° 2Θ and a measuring time of 2 s per step. To get a good resolution of the (002) peak of kaolinite and the (004) peak of chlorite, we analysed the samples also in the range 27.5–30.6° 2Θ with a step size of 0.01° 2Θ and a measuring time of 4 s per step. The percentages of smectite, illite, chlorite and kaolinite were calculated based on their peak areas and empirically estimated weighting factors^36^,^37^.

### Radiocarbon dating

Planktic and calcareous benthic foraminifera were picked from the sand fraction for AMS ^14^C dating (Table 2), and the resulting ages were previously published^21^. The upper three ages overlap within analytical error and span within the error ranges 8.1–8.4 cal. ka BP. All dates were obtained from the same lithological Subunit 1.2 (Fig. 3). Only the oldest age of 8.6 cal. ka BP was used for calculating the sedimentation rates applied to the pore water model.

### Rock magnetic measurements

Samples for rock magnetic measurements were continuously (sampling interval ∼2.5 cm) taken in 2.2 cm × 2.2 cm × 1.8 cm plastic cubes. The discrete samples were analysed at the palaeomagnetic laboratory at the Department of Geosciences, University of Bremen. Acquisition curves of isothermal remanent magnetization (IRM) were measured on a cryogenic rock magnetometer (model 2G Enterprises 755HR) utilizing its in-line pulse magnetizer for d.c. fields of up to 700 mT. In addition, an IRM subsequently acquired in oppositely directed fields of 100 mT (IRM_–100_) and 300 mT (IRM_–300_) was measured to estimate the ratio of low- ([titano-]magnetite/maghemite) to high-coercive (haematite, goethite) magnetic minerals by calculation of S_−100_=IRM_−100_/IRM_700_ and S_−300_=IRM_−300_/IRM_700_ ratios^38^. S_−100_ values close to −1 indicate high amounts of low-coercive magnetic minerals (magnetite) while values close to 0 or even +1 represent high amounts of fine-grained (titano-)magnetite/maghemite and/or haematite or goethite, respectively. S_−300_ values close to 1 indicate magnetic mineral assemblages dominated by (titano-)magnetite/maghemite, while lower values reflect increasing fractions of haematite or goethite^39^.

### X-ray fluorescence core scanning

After splitting, the archive halves of the sediment cores were scanned with an Avaatech XRF-core scanner^40^ with a sampling interval of 1 cm and spot size of 1 cm^2^. To demonstrate the variation of Fe to other elemental peak counts the log-normalized ratio of Fe counts to the sum of Ti, Zr, K and Si counts was calculated^41^ (Fig. 3).

### Measurements of organic and inorganic carbon and nitrogen

Total carbon and TN were analysed on freeze dried and milled bulk samples (sampling interval 10 cm) with an Elementar Vario EL III. TOC contents were determined after removal of the total inorganic carbon (TIC, carbonates) with HCl using an ELTRA CS-2000.

### Porosity measurements

Porosity calculations were carried out from Multi-Sensor Core Logger (MSCL) gamma ray attenuation measurements (sample interval 1 cm) converted to wet bulk density (WBD) and from discrete sediment sample water content and density measurements (sampling interval 10 cm) after Niessen *et al*.^42^. The MSCL porosity was calculated using equation (1).




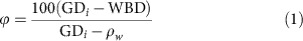




where 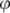 is porosity (vol%), GD_*i*_ is interpolated grain density from discrete samples, WBD is wet bulk density from MSCL and *ρ*_*w*_ is pore water density. We have assumed a constant *ρ*_*w*_ of 1.03 g cm^−3^ because pore water density for *in situ* conditions differs insignificantly (0.99–1.055 g cm^−3^)^42^. GD was calculated from measurements of water content (*w,* weight%) and density of the milled sediment (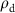, Micromeritics gas-pycnometer AccuPyk 1330). Mean GD from 105 samples is 2.66 g cm^−3^ (min. 2.57, max. 2.74 g cm^−3^). The calculation is:




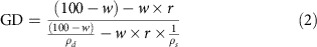




where *ρ*_*s*_ is density of salt (2.1 g cm^−3^) and *r* is the mass ratio of salt to water (=0.036, at 3.5% salinity).

### Chloride concentrations

Pore water was retrieved from the work halves of the sediment cores by means of rhizons with an average pore diameter of 0.15 μm (Rhizosphere Research Products, Wageningen) according to the procedure described by Seeberg-Elverfeldt *et al*.^43^ and Dickens *et al*.^44^. Chloride concentrations (mmol^−1^=mM) were determined by means of ion chromatography (Metrohm IC Advanced Compact 861) at a flow rate of 0.7 ml min^−1^ on pore water aliquots which were diluted 1:50 with deionized water. Before pore water sampling and analysis the cores had been stored wrapped in cling film and sealed in plastic D-tubes at 4 °C for about 6 (PS69) and 3 (PS75) years. We assume that artefacts produced by potential evaporation of pore water during sediment storage are negligible. In case evaporation had taken place, any measured pore water freshening should thus represent a minimum estimate of the ‘real’/‘*in situ*’ chloride anomaly.

### Diffusion-advection model for chloride

Chloride concentrations were modelled using a diffusion-advection equation^45^,^46^ (equation (3)) which takes into account the influence of porosity on burial velocities and tortuosity and the dependence of the diffusion coefficient on temperature^46^,^47^. No indication of mixing by bioturbation was detected in the lower part of the sediment core. Therefore, the equation does not include a term describing the effect of bioturbation. Some model runs were carried out assuming a 10 cm bioturbated layer and bioirrigation in more recent times (<4.1 cal. ka BP) but these runs did not significantly affect the chloride profiles.




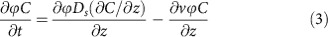




where *C* is concentration, *φ* is porosity, *t* is time, *v* is the pore water velocity (equation (4)), *D*_*s*_ is the temperature-corrected diffusion coefficient for chloride in the sediment (equation (5)) and *z* is depth (model parameters listed in Supplementary Table 1).

The pore water velocity in the sediments was calculated under the assumption that water mass is conserved and that, at depth, the solid-phase and pore water velocities advect at the same velocity^48^:




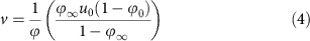




where the *u* refers to the burial velocity of solids, subscript 0 refers to the parameter at the sediment–water interface and subscript ∞ refers to the parameter at great depth.

The solid phase velocity is likewise calculated assuming conservation of solid mass:




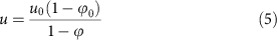




The diffusion coefficient for chloride in the sediment is hindered by the presence of grains through the geometric tortuosity (*θ*):




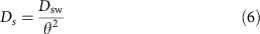




where *D*_sw_ is the chloride diffusion constant in sea water. *D*_sw_ (in cm^2^ year^−1^, equation (7)) was calculated assuming a linear dependency with temperature^47^:




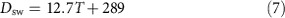




where *T* is the temperature in °C which increases with depth from a value of 0 °C at the sediment surface according to a geothermal gradient of 25 °C km^−1^. The tortuosity can be correlated to the formation factor (*F*), which represents the ratio of electrical resistivity in pure water over the resistivity of the sediment times the porosity^49^:




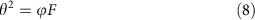




A compilation of formation factor and porosity measurements from Antarctic sediment core sites^50^ reveals that the porosity-formation factor relation can be established via an exponential equation (equation (9), Supplementary Fig. 2). The best fit for parameters *α*_*1*_ and *α*_*2*_ was determined by using a least-squares regression.




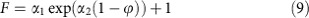




The porosity profile (Supplementary Fig. 3) was modelled independently for four depth intervals (Supplementary Table 2), assuming compaction algorithms that varied either exponentially (equation (10)) or double exponentially (equation (11)) with depth.




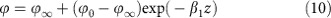







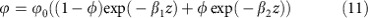




where *β* is the depth-attenuation factor and *ϕ* the ratio of influence of various depth-attenuation factors. Equation (10) was solved using a Crank–Nicholson scheme with a 0.05 time step, a 0.5 cm spatial discretization and a total model domain of 6,000 cm.

Several model runs with a direct change from limnic to marine chloride concentrations at different times and sediment depths were conducted for site PS69/288, but led to imperfect fitting between measured and modelled chloride concentrations (Supplementary Fig. 4). Only a limnic–brackish–marine model run led to a good fit between measured and modelled concentrations (Fig. 5, Supplementary Movie 2). Fresh pore water composition was also detected in four other cores from Pine Island Bay that displayed Unit 2 or Unit 1 type lithofacies at their bottom (Fig. 1b, Table 1). Pore water samples from five other cores did not show signs of freshwater in grounding zone proximal sediments (Fig. 1b, Table 1). Because low chloride concentrations were found in the sediment cores from the seafloor basins only, we conclude submarine groundwater discharge^51^ or other sources of freshwater in Pine Island Bay to be negligible. We also rule out freshwater supply by clay mineral or opal dewatering because temperatures of >80 °C and >55 °C, respectively, are required for these dehydration processes^20^,^52^.

The decrease of the chloride concentration with sediment depth evident from the profiles arises from the interposition and diffusive exchange of chloride during the deposition of the limnic, brackish and marine stages. Kinks in the simulated profiles occur at the boundaries of the various stages due to the differential compaction of the sediment layers, especially within the low-porosity layers that characterize the brackish sediments. These brackish layers inhibit chloride diffusion-advection from the marine stage, and must have been well compacted with low porosities during the brackish–marine transition to maintain the kink in the chloride profile up to the present day.

### Data availability

The data that support the findings of this study are available in PANGAEA, Data Publisher for Earth & Environmental Science, at https://doi.pangaea.de/10.1594/PANGAEA.873755 with the identifier (doi: 10.1594/PANGAEA.873755)^53^, and within Supplementary Information files associated with this manuscript.

## Additional information

**How to cite this article:** Kuhn, G. *et al*. Evidence for a palaeo-subglacial lake on the Antarctic continental shelf. *Nat. Commun.* **8,** 15591 doi: 10.1038/ncomms15591 (2017).

**Publisher’s note:** Springer Nature remains neutral with regard to jurisdictional claims in published maps and institutional affiliations.

## Supplementary Material

Supplementary Information Supplementary Figures, Supplementary Tables and Supplementary References

Supplementary Movie 1 Diffusion-advection model for site PS69/288 describing the evolution of chloride concentrations in pore water trough time. Modelled profiles of chloride concentrations in pore water are given from 11,200 yrs. BP until recent 0 yrs continuously. The modelled modern profile (= 0 yrs. BP) matches the measured chloride concentrations in the core (black squares), for details, see Methods.

Supplementary Movie 2 Variable sill depth. Extent of subglacial water in the vicinity of site PS69/288 (blue: areas covered by subglacial water; green, yellow, orange and red: areas covered by grounded ice) assuming a lake level increase during deglaciation (reference to modern water depth). Animated "flooding"of the lake area.

## Figures and Tables

**Table 1:** Stations with strongly reduced and only slightly reduced chloride content in pore water at the basis of sediment cores.

**Station**	**Gear**	**Low Cl** ^−^ **(mM)**	**Latitude S**	**Longitude W**	**Water depth (m)**	**Core recovery (m)**
PS69/288-2	GBC	—	74° 24.91′	102° 59.52´	773	0.44
PS69/288-3	GC	345	74° 24.94′	102° 59.48´	772	9.12
PS69/295-1	GC	491	74° 28.73′	104° 06.09´	1,151	4.86
PS75/159-1	GC	477	74° 47.99′	102° 21.31'	1,046	8.62
PS75/166-3	GC	478	74° 36.28′	106° 38.59'	1,385	7.44
PS75/214-1	GC	360	74° 31.96′	102° 37.28'	641	7.72
						
		**High Cl** ^−^ **(mM)**				
PS69/291-1	GC	526	74° 41.17′	104° 09.51'	1,023	9.86
PS69/292-2	GC	536	74° 40.92′	105° 11.60'	1,407	6.83
PS69/296-1	GC	512	74° 21.41′	104° 45.08'	1,433	9.09
PS75/167-1	GC	522	74° 37.37′	105° 48.11'	526	9.34
PS75/168-1	GC	511	74° 36.71′	105° 51.96'	652	7.44

GBC, giant box core; GC, gravity core.

Marine chloride (Cl^−^) content is about 560 mM.

**Table 2:** Radiocarbon age control.

**Core**	**Publi. code BETA-**	**Composite core depth (cmbsf)**	**Material dated**	**Conv.** ^**14**^ **C age yrs BP**	**±1** ***σ***	**MRE ±100years**	**Cal. yr BP 1** ***σ***		**Cal. yr BP mean**
							**Min**	**Max**	
PS69/288-3 GC	338486	324	pF, bF	8,740	50	1,300	8,177	8,402	8,290
PS69/288-3 GC	337427	344	pF, bF	8,640	40	1,300	8,101	8,330	8,216
PS69/288-3 GC	331574	404	pF, bF	8,720	40	1,300	8,171	8,383	8,277
PS69/288-3 GC	337428	464	pF, bF	9,020	40	1,300	8,447	8,749	8,598
PS75/214-1 GC	300846	480+497	F	11,090	50	1,300	11,042	11,331	11,187

Uncorrected and calibrated AMS ^14^C dates on calcareous microfossils together with sample depth and dated material (bF, benthic foraminifera; F, mixed benthic and planktic foraminifera; pF, planktic foraminifera). A marine reservoir effect (=MRE) correction of 1,300±100 years was applied^55^. The corrected ^14^C-dates were calibrated with the CALIB Radiocarbon Calibration Program version 7.0.2 (http://calib.qub.ac.uk/calib/) using the MARINE09 calibration data set. We show the entire 1σ range for each calibrated age (Min, Max), but quote the mean age throughout the text. The upper three ^14^C-dates plot all between 8.1 and 8.4 cal. ka BP within their Min–Max 1*σ* ranges. The lowermost age of 8.6 cal. ka BP is the oldest and most reliable (minimum) age for the onset of full marine conditions at this core position. The AMS ^14^C dating was carried out at BETA Analytic Inc., Miami, Florida, USA. Dates from cores PS69/288-3 GC and PS75/214-1 GC have been previously published in Smith *et al*.^21^ and Hillenbrand *et al*.^56^, respectively. For this study we used and recalibrated the oldest date from core PS75/214-1 GC (no composite depth), which provides the minimum age for grounded ice retreat from inner Pine Island Bay following the LGM^21^,^56^.
