# Supplementary material for: Evidence for a palaeo-subglacial lake on the Antarctic continental shelf
Source: Nat Commun. 2017 Jun 1;8:15591. doi: 10.1038/ncomms15591 (PMC5461483; doi:10.1038/ncomms15591)
Supplement: Supplementary Information — Supplementary Figures, Supplementary Tables and Supplementary References [file ncomms15591-s1.pdf]

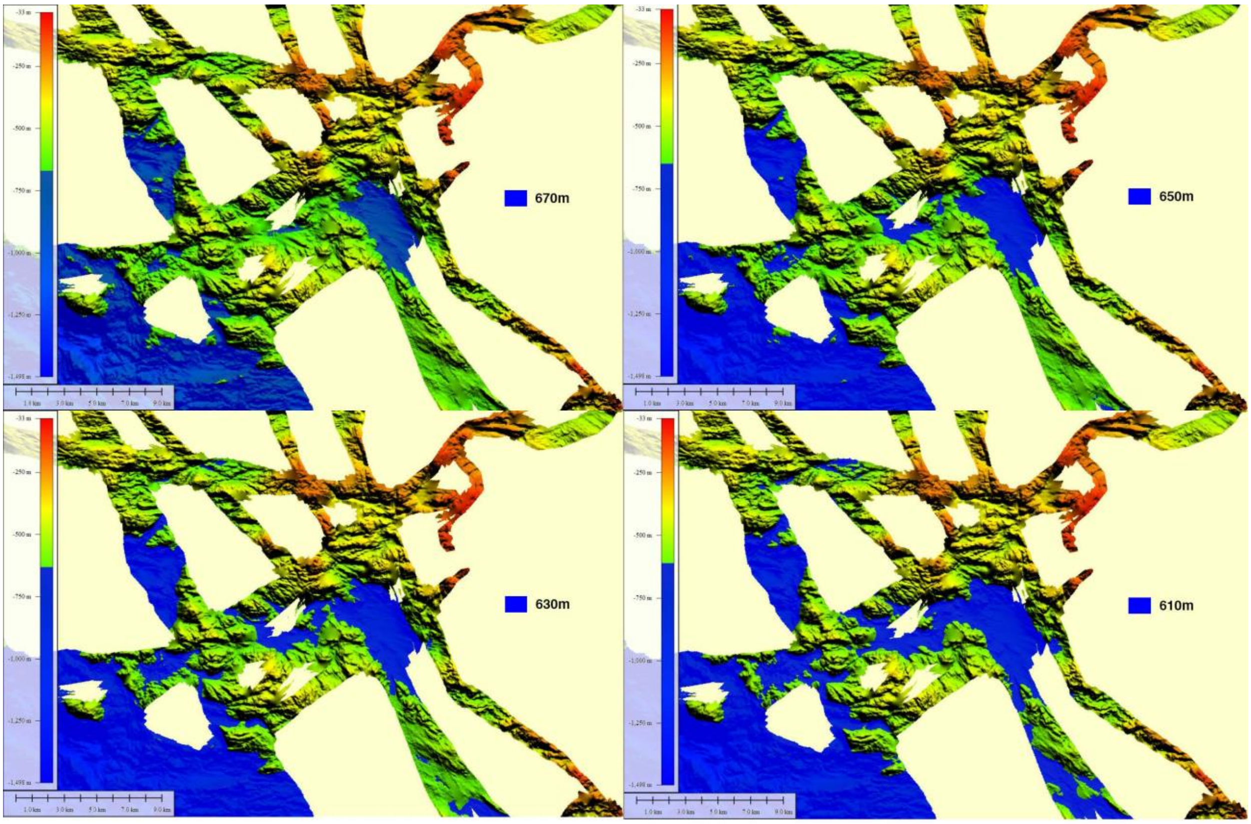

Supplementary Figure 1 | Variable sill depth. Extent of subglacial water in the vicinity of site PS69/288 (blue: areas covered by subglacial water; green, yellow, orange and red: areas covered by grounded ice) assuming a lake level/ice shelf base at 670 m, 650 m, 630 m and 610m of modern water depth (see as well animated graphic "sub-ice cavern flooding" as Supplementary Movie 2).

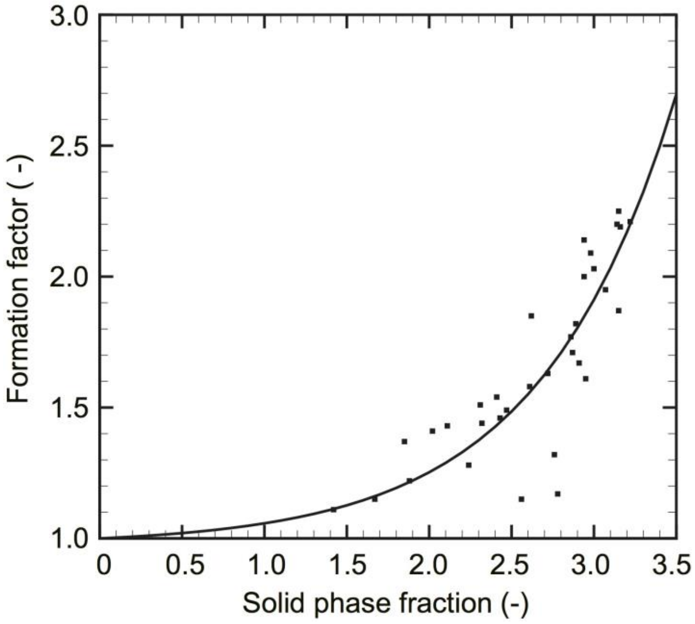

Supplementary Figure 2 | Formation factor measured in Antarctic marine sediments (based on data from Schlüter et al.)[1].

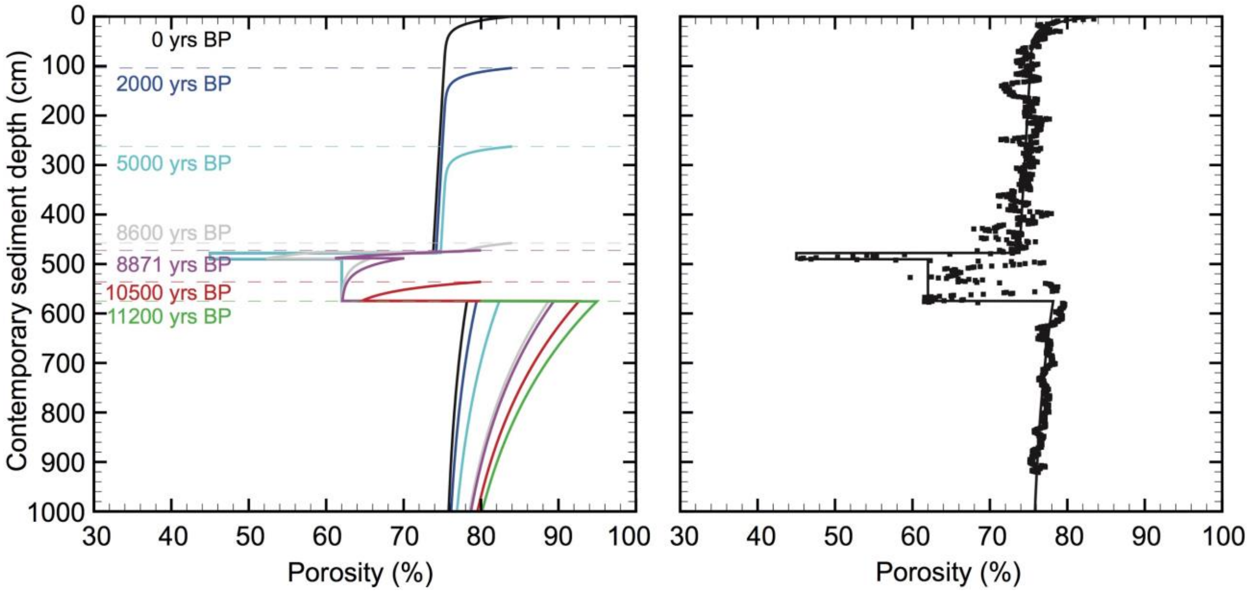

Supplementary Figure 3 | Porosity evolution. The evolution of porosity at site PS69/288 vs. time plotted as a function of the contemporary sediment depth (left). Horizontal dashed lines indicate the palaeo-sea floor at particular times at the core site. The simulated profile for 0 cal. yr BP (black line) is compared to the measured data (black dots) (right).

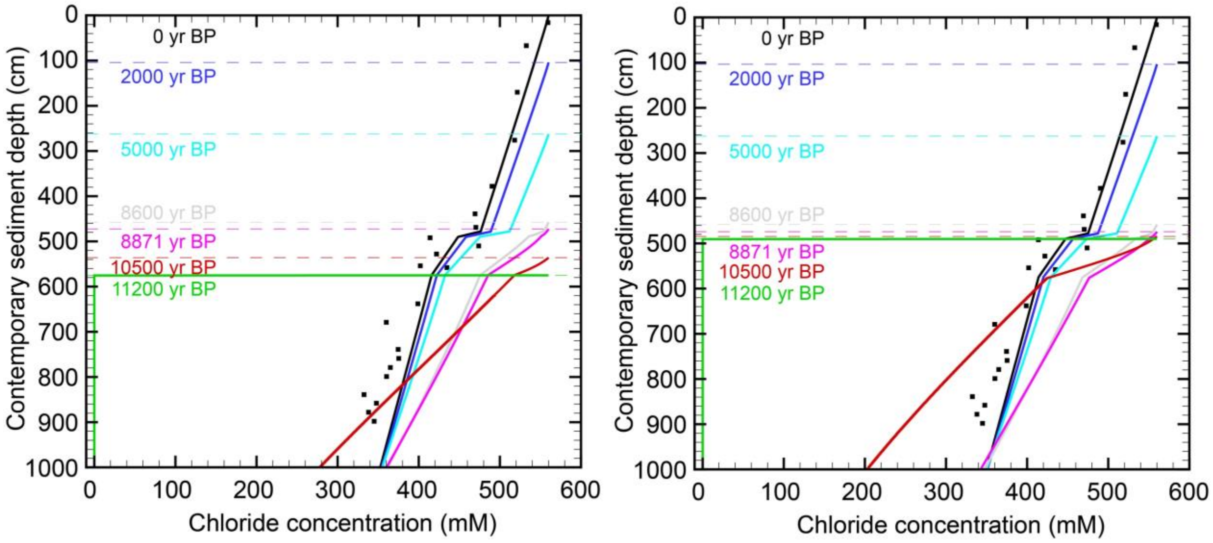

Supplementary Figure 4 | Diffusive-advective chloride models. Diffusive models without a brackish phase for a limnic-marine transition at 575 cm below sea floor (cmbsf; left) and 11,200 yrs. BP and at 490 cmbsf (right). Final model results (black line) do not fit with measured concentrations (black squares). Only the insertion of a brackish period makes the data fit (Fig. 5, and animated graphic file Fig, 5 as Supplementary Movie 2).

Supplementary Table 1 | Model parameters (bsf = below sea floor)

Name	Symbol	Unit	Value	Remark
Porosity at great depth	φ_∞	-	0.75	From bottom layer
Formation factor constant 1	α_1	-	0.02425	Fitted
Formation factor constant 2	α_2	-	12.177	Fitted
Temperature	T	^{o}C	0	Assumed
Burial velocity of solids at sea floor, brackish stage (11200 – 8871 cal. yrs. BP)	u_0	cm/y	0.08445	Fitted from chronological data
Burial velocity of solids at sea floor, marine stage (8871 – 0 cal. yrs. BP)	u_0	cm/y	0.0835	Fitted from chronological data
Concentration at sea floor (marine stage 8.6 – 0 cal. ka BP)	C_0	mM	560	Assumed
Concentration at sea floor (brackish stage 11.2 – 8.6 cal. ka BP)	C_0	mM	200	Assumed
Concentration gradient at great depth	$\partial C/\partial z_\infty$	mol m^{-4}	0	Imposed at 6,000 cm

Supplementary Table 2 | Parameters used in the 4-layer compaction model

Type	Unit (and Subunit)	Top depth (cmbsf)	φ_0 (-)	β (cm^{-1})	ϕ (-)
Double Exponential	1	0	0.84	0.08, 0.00005	0.9
Exponential	2.1	478	0.8	0.05	0.45
Exponential	2.2	490	0.8	0.05	0.62
Exponential	3	575	0.95	0.0032	0.75

Supplementary References

1 Schlüter, M., Rutgers van der Loeff, M. M., Holby, O. & Kuhn, G. Silica cycle in surface sediments of the South Atlantic. *Deep Sea Research Part I: Oceanographic Research Papers* **45**, 1085-1109, (1998).
