# Supplementary figures and images for: Evidence for a palaeo-subglacial lake on the Antarctic continental shelf
Source: Nat Commun. 2017 Jun 1;8:15591. doi: 10.1038/ncomms15591 (PMC5461483; doi:10.1038/ncomms15591)

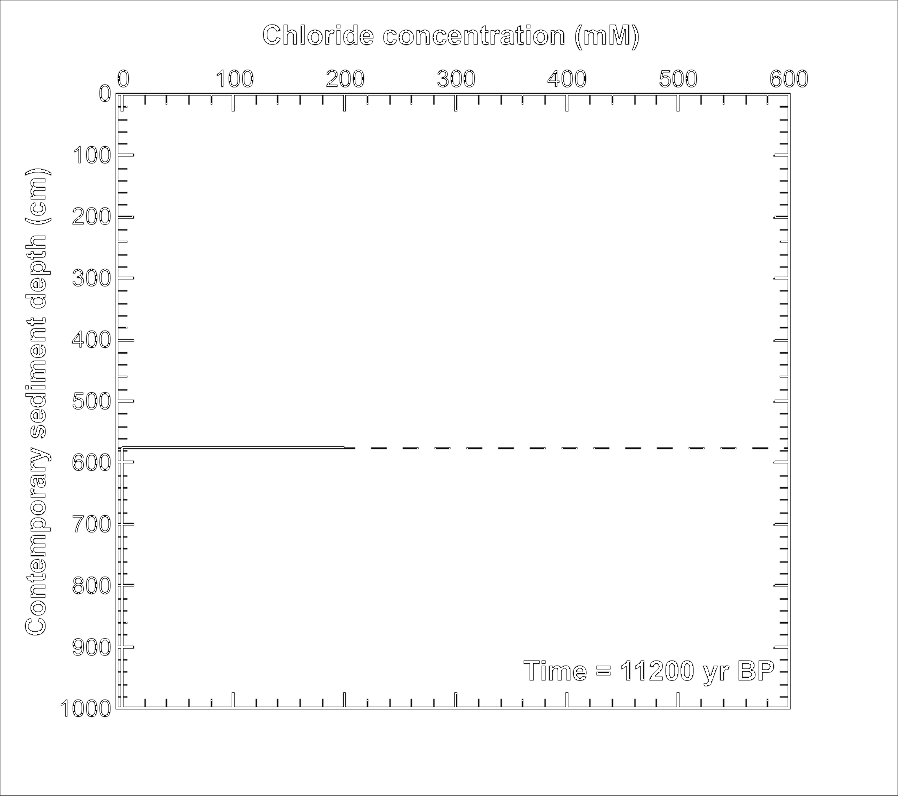

Supplement: Supplementary Movie 1 — Diffusion-advection model for site PS69/288 describing the evolution of chloride concentrations in pore water trough time. Modelled profiles of chloride concentrations in pore water are given from 11,200 yrs. BP until recent 0 yrs continuously. The modelled modern profile (= 0 yrs. BP) matches the measured chloride concentrations in the core (black squares), for details, see Methods. [file ncomms15591-s2.tif]

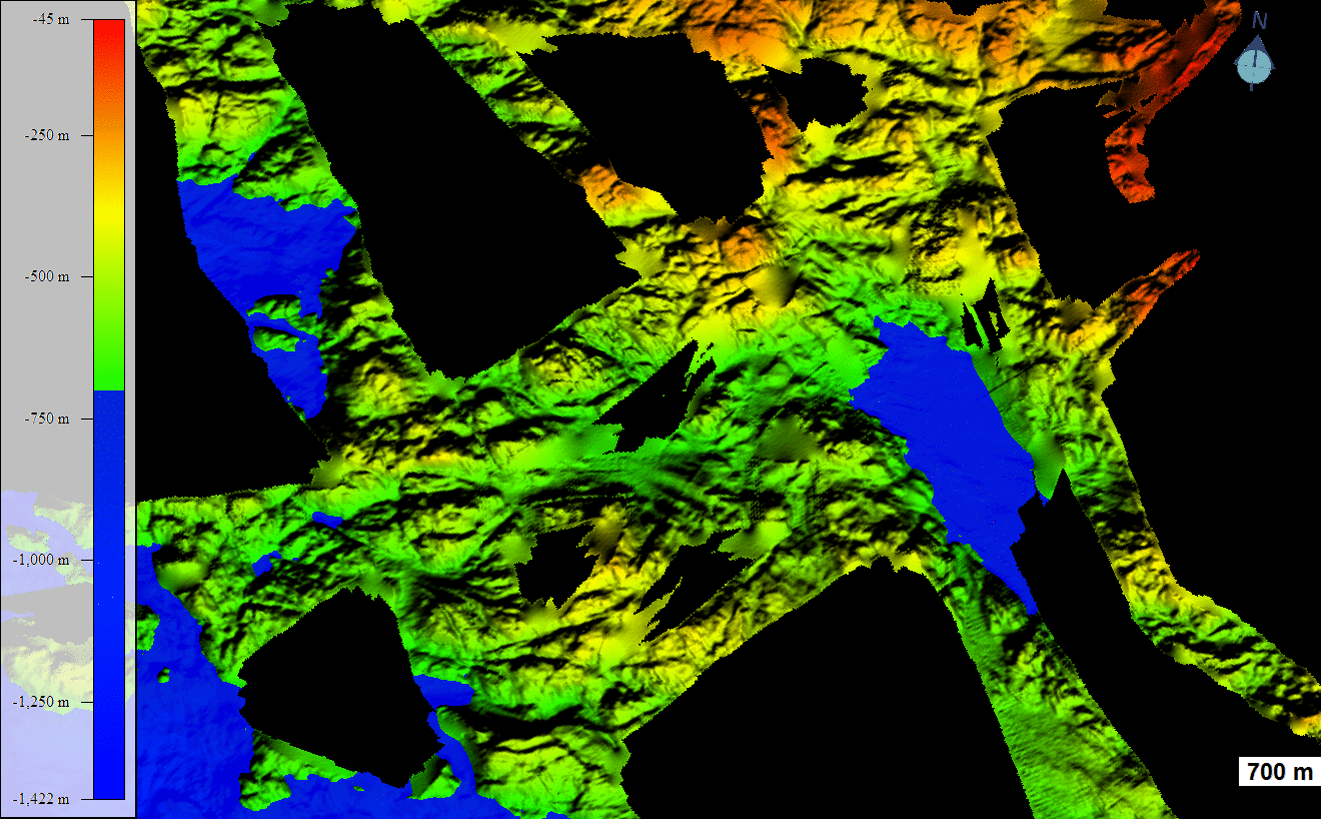

Supplement: Supplementary Movie 2 — Variable sill depth. Extent of subglacial water in the vicinity of site PS69/288 (blue: areas covered by subglacial water; green, yellow, orange and red: areas covered by grounded ice) assuming a lake level increase during deglaciation (reference to modern water depth). Animated "flooding"of the lake area. [file ncomms15591-s3.tif]
